# Use of TNF Inhibitors in Rheumatoid Arthritis and Implications for the Periodontal Status: For the Benefit of Both?

**DOI:** 10.3389/fimmu.2020.591365

**Published:** 2020-10-23

**Authors:** Fatima Zamri, Teun J. de Vries

**Affiliations:** Department of Periodontology, Academic Centre for Dentistry Amsterdam (ACTA), University of Amsterdam and Vrije Universiteit, Amsterdam, Netherlands

**Keywords:** Osteoclast (OC), TNF - α, inflixmab, etenarcept, Rheumatoid arthritis, Periodontitis

## Abstract

The inflammatory diseases rheumatoid arthritis (RA) and periodontitis show similarities in misbalances of cytokine levels, such as tumor necrosis factor-α (TNF-α). RA has been treated for two decades with TNF inhibitors which are effective by blocking TNF’s destructive action. Since RA and periodontitis show similarities in high levels of TNF, the periodontal status of RA patients may improve with the use of anti-TNF therapy. To assess this, a systematic review with special emphasis on duration of therapy was performed to evaluate the effect of anti-TNF-α treatment on the periodontal status of RA patients. Overall, studies showed an improvement in periodontal health with anti-TNF therapy. When analyzed over time (6 weeks to 9 months), it became apparent that initial improvements concerned bleeding on probing (BOP) and gingival index (GI) after therapy duration of 6 weeks. Periodontitis parameters that improved after prolonged treatment were: probing pocket depth (PPD) after 3 months and clinical attachment level (CAL) after 6 months. In conclusion, this systematic review reveals that anti-TNF treatment is therefore not only beneficial for rheumatic joints but also for the gums of rheumatoid arthritis patients. We propose that the sequential tissue recovery due to anti-TNF therapy progresses as follows: 1. block of diapedesis by lowering vessel permeability, 2 fewer leukocytes in the inflamed tissue, and 3. reduced proteolytic activity and subsequent repair of collagen fiber functionality and normalization of osteoclast activity. Clinically, this could lead to a decrease in bleeding on probing and ultimately in an improved clinical attachment level.

## Introduction

Both periodontitis and rheumatoid arthritis are inflammatory diseases. Periodontitis is caused by an inflammatory response to microbes and bacterial toxins, eventually leading to destruction of supporting periodontal tissues around the teeth (1). Rheumatoid arthritis (RA) manifests primarily as a persistent synovial inflammation which causes damage to articular cartilage. If not treated in time, the underlying bone is also affected (2). There are quite a few similarities between RA and PD. Both diseases show similarities in the prevailing cytokines within the tissues. Overexpression of TNF is at stake in both diseases and causes an imbalance in cytokine levels and therefore damage of soft tissues, progressing to bone, where osteoclasts are further activated by TNF-α (2–4). Furthermore RA patients are prone to develop periodontitis, possibly due to an increase of circulating TNF levels and/or deteriorated motor skills needed for oral hygiene maintenance as a result of damage in the joints. On the other hand, the relationship could also be reverse: periodontitis could cause inflammation of joints induced by periodontal pathogens that enter the circulation due to periodic and frequent oral bleeding. Periodontal pathogens have been detected in inflamed joints of RA patients (5, 6).

Tumor necrosis factor alpha (TNF-α, from here the common term TNF will be used) is a pro-inflammatory cytokine produced in occurrence of inflammation by cells like macrophages and monocytes (7). It is a hallmark cytokine of the pro-inflammatory immune response. TNF can bind to two different receptors, namely TNFR1 and TNFR2 (p75 TNFR). Binding to each receptor sets different signaling cascades in motion that can lead to apoptosis, differentiation, proliferation and migration of cells causing an inflammatory reaction (8). On the other hand TNF plays an inducing role in bone resorption by attributing to the receptor activator of Nf-kB Ligand (RANKL)-signaling pathway by directly activating osteoclast precursor cells (9, 10). TNF sets a natural immune response in motion in reaction to an infection. However in high concentrations it can cause side effects such as a non-tempered inflammatory reaction, increase in osteoclast precursors and osteoclast formation resulting in bone resorption (11–14). Levels of TNF are associated with less favorable indices of the periodontal parameters such as bleeding on probing (BOP), probing pocket depth (PPD), and clinical attachment level (CAL) (15).

TNF inhibitors are used clinically to counterbalance the high TNF levels accounting for joint inflammation, hereby preventing TNF tissue damage in RA. TNF inhibitors have been available for inflammatory diseases like rheumatoid arthritis, ankylosing spondylitis, psoriatic arthritis, psoriasis, ulcerative colitis and Crohn’s disease since 1998. The presently available TNF inhibitors are infliximab, adalimumab, golimumab and etanercept and certolizumab pegol. These inhibitors have an immunoglobin (Ig) structure in common. Infliximab has a 65% similarity to human IgGs, and golimumab and adalimumab have the highest similarity with human IgG. Etanercept is a recombinant fusion drug existing of TNF p75 receptor and the fc component of human IgG1. Anti-TNF reduces inflammatory reaction of the body by blocking TNF-α, hence preventing it to bind on its receptor (TNFR1 or TNFR2). However a prolonged use of TNF inhibitors has a few possible side effects like: hepatotoxicity, malignity, greater risk for infection, immunogenicity and cutaneous reactions.

Heart failure is a contraindication for anti-TNF therapy (16). High levels of TNF- α are correlated with cardiac remodeling resulting eventually in disruption of the cardiac function (17, 18). Therefore high TNF-alfa concentration is a risk for heart failures. Use of TNF inhibitors cause a decrease in TNF- α levels and can so prevent cardiac remodeling and heart failures. Recent studies have shown that TNF inhibitors can have this protective effect (18). However, various studies have shown that cardiac function can be disturbed in patients on TNF inhibitors and are therefore a contraindication in patients with heart failure. To have a better understanding of the effect of the possible protective effect of TNF inhibitors in cardiovascular disease more studies are required.

In vitro and in vivo studies have already proven that the use of anti-TNF (infliximab and etanercept) inhibits osteoclast formation (12, 13, 19, 20). Since periodontitis is manifested as breakdown of alveolar bone, treatment with anti-TNF might have similar positive effects for the periodontium as for the inflamed joints of RA patients. For periodontitis, its effect could be two-fold: lowering of soft-tissue break-down and an inhibition of bone degradation.

Previous work summarizing the effectiveness of anti-TNF therapy in RA and ankylosing spondylitis (AS) has suggested a a positive spin-off for the periodontium (1, 21, 22). No study thus far, however, has assessed how the duration of anti-TNF therapy improves the specific periodontal parameters. The aim of this study is specifically to assess the effectiveness of anti-TNF treatment on possible improvement of specific periodontal parameters. With such a novel approach, we hope to link the blocking of TNF to clinical and therefore biological events at the level of the periodontal tissues.

## Methods

### Literature Search

For the literature search a methodological approach was adopted by using PRISMA (Preferred Reporting Items for Systematic Reviews and Meta- Analyses). This method was used to reduce bias in literature selection by the authors.

The electronic search was carried out in the following databases: Embase, Pubmed and Web of Science. Studies up to January 2020 and publications in English were included in this search. The keywords used for the search were: “periodontitis,” “periodontal diseases,” “periodontal,” “adalimumab”, “etanercept”, “infliximab”, “certolizumab pegol”, “antibodies tumor necrosis factor- alpha,” and “TNF-alpha inhibitors” (**Table 1**), including the search filters ‘Humans’ and ‘English’ resulted in excluding suitable publications; therefore, these filters were not used. However, further on we restricted our search to articles written in English only.

**Table 1:** Search query TNF-alpha inhibitors.

Search	Query
#1	Periodontitis OR periodontal diseases OR periodontal
#2	Adalimumab OR etanercept OR infliximab OR certolizumab pegol OR antibodies tumor necrosis factor- alpha OR TNF-alpha inhibitors
#3 (#1 AND #2)	(periodontitis OR periodontal diseases OR periodontal) AND (adalimumab OR etanercept OR infliximab OR certolizumab pegol OR antibodies tumor necrosis factor alpha OR TNF-alpha inhibitors)

#### Screening and Selection

To select suitable publications a set of inclusion and exclusion criteria was used. All in vitro and animal studies were excluded. Cohort studies, case reports, randomized controlled trials, and longitudinal studies in English were included. Furthermore, only studies that tested at least one of the clinical periodontal parameters (probing depth, BOP, and clinical attachment loss) or radiographic parameters were included. Outcomes of clinical trials compared the effect of anti-catabolic medication on positive or negative effects on the periodontium. Articles that did not meet this outcome were excluded.

All titles and abstracts of the publications from the electronic search were screened by two reviewers (FZ and TV) and discussed. When mutual agreement between the two reviewers could not be reached or when the suitability of the publication was questionable the full article was examined by both. The selected literature was divided into the different follow-up periods and presented in tables summarizing the effect of the medications on the following periodontal parameters: plaque index (PI), gingival index (GI), BOP, PPD, and CAL. PI is a score where supragingival plaque is recorded on four or six sites of each tooth. The score defines the absence or presence of plaque by marking it with a positive or negative score. Accumulation of these scores finally results in the PI, which varies from 0 till 100%. The GI can be used to evaluate the condition of the gingiva and is scored at four sites around the teeth. The score varies from 0 to 3, a score of 0 for a normal gingiva and 3 for a severe gingival inflammation (23). BOP is the percentage of bleeding points around the teeth. It is scored at 6 sites around the tooth and can vary from 0 to 100%. PPD is the measurement from the gingival margin to the bottom of the pocket. A measurement of more than 3 mm indicates clinical attachment loss. CAL is defined as the distance from the cemento-enamel junction (CEJ) to the bottom of the pockets.

## Results

**Figure 1:**
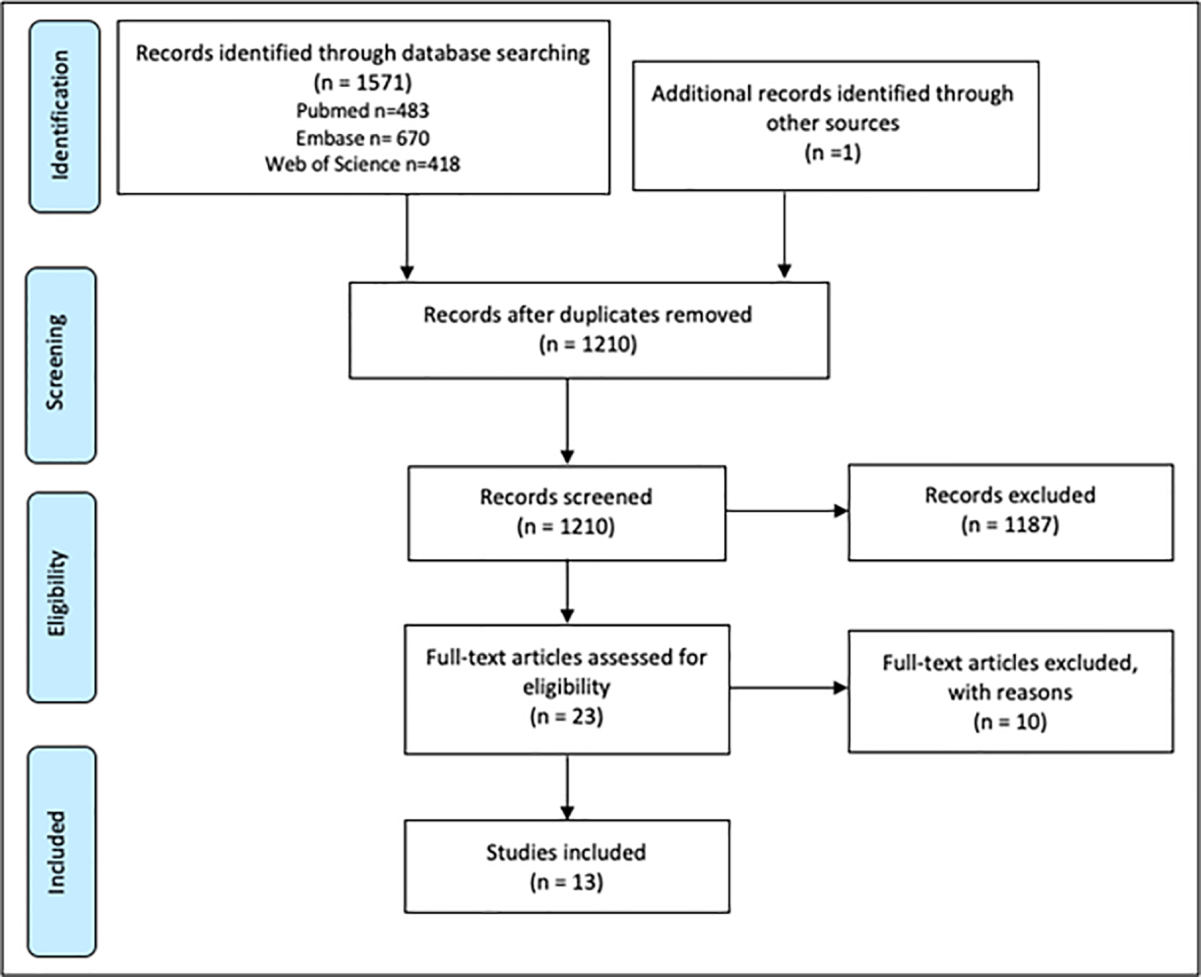
Literature search, strategy, and results.

The electronic research that was carried out resulted in a total of 1,571 articles. After manually removing the duplicates a total of 1,209 publications remained. An additional 1 record was found through manually screening reference lists of articles that were found. All the 1210 articles were manually screened for suitability by reading the titles and abstracts. This resulted in a full text read of 23 articles of which 10 publications were excluded with reasons like: no full text available, no clear description of the used materials and methods, or use of new medication during follow-up period. Finally, 13 publications were included in this review (**Figure 1**). Results and methods of publications discussing the effect of anti-TNF therapy on periodontal parameters (PI, GI, BOP, PPD, and CAL) are presented in **Table 1**. Summarization is in chronological order of publication and was divided in smaller groups depending on the follow-up period. In order to provide a complete impression, tables will also show when examiners in the study were blinded or calibrated.

### The Effect of Anti-TNF Treatment in Case-Control Studies

The first group of studies that will be discussed is the group with no follow-up assessment which consisted of 3 studies (**Table 2**). Studies in this group compared patients on anti-TNF therapy to control groups, without baseline assessment before start of anti-TNF therapy. Mayer et al. (24) compared periodontal parameters in RA patients with anti-TNF therapy (mean of 26 months) and in RA patients without therapy. GI, BOP, and CAL were significantly lower in patients using infliximab. When RA patients on anti-TNF therapy (mean of 26 months) were compared to a control group, patients with periodontal disease, without RA or another systematic inflammatory disease and no anti-TNF therapy, BOP and CAL showed also significantly lower parameters. Mayer et al. (25) conducted a similar study with infliximab where RA patients using anti-TNF therapy (RA+) were compared with patients having autoimmune diseases (rheumatoid arthritis, psoriatic arthritis, and systemic sclerosis). The RA+ group showed a statistically significant decrease in periodontal parameters GI, BOP, and PPD. This study also made a comparison between RA patients that used anti-TNF therapy (RA+) and healthy subjects. Also the comparison was made with patients without RA or any other autoimmune diseases. This study showed that there was no statistically significance between the groups in PI, GI, BOP, and PPD (25). Schiefelbein et al. studied the effect of anti-TNF by comparing RA patients that have been using anti-TNF for at least 12 months with non-RA patients. Patients using anti-TNF had a significant lower BOP. CAL showed no statistically significant change (26).

**Table 2:** Studies assessing the effect of TNF inhibitors on periodontal parameters.

Authors	TNF inhibitor	Subjects	Assessment	PI	GI	BOP	PPD	CAL
**Varied, no baseline**								
Mayer et al. (24)	IFX	RA+ (n = 10) compared to RA without anti-TNF-α (n = 10)	1 moment (1 calibrated examiner)	NS p > 0.05	↓ (sig.) p = 0.0042	↓ (sig.) p = 0.0146	NS p = 0.0554	↓ (sig.) p = 0.0273
		RA+ (n = 10) compared to healthy patients (n = 10)	1 moment(1 calibrated examiner)	NS p > 0.05	NSp > 0.05	↓ (sig.) p = 0.0146	NS p = 0.0554	↓ (sig.) p = 0.0273
Mayer et al. (25)	IFX	RA+ (n = 12) compared to AI (RA, PA and SSc) (n = 36)	1 moment(2 calibrated examiners)	NS p = 0.0548	↓ (sig.) p = 0.0005	↓ (sig.) p = 0.0002	↓ (sig.) p = 0.0001	–
		RA+ (n = 12) compared to healthy patients (with PD, no AI’s or anti-TNF-α) (n = 12)	1 moment(2 calibrated examiners)	NS p > 0.05	NSp > 0.05	NSp > 0.05	NS p > 0.05	–
Schiefelbein et al. (26)	ETN (n = 2)ADA (n = 5)Golimumab (n = 6)	RA with PD on anti-TNF-α for >12 months (n = 13) versus non RA with PD (n = 13)	1 moment(1 calibrated examiner)	NSp = 0.182	–	↓ (sig.)p = 0.045	↓ (NS)p = 0.068	NSp = 0.134
**30 days follow up**								
Üstün et al. (27)	IFX (n = 9)ADA (n = 7)	RA patients with PD (n = 10)	BL + after 30 days(1 examiner)	NS p = 0.779	↑(sig.) p = 0.016	NS p = 0.067	NS p = 0.413	NSp = 0.326
**6 weeks follow up**								
Ortiz et al. (28)	IFXETNADA	RA patients on PDT and anti-TNF-α (n = 10)	BL + after 6 weeks(1 calibrated examiner)	↓ (sig.)p < 0.01	↓ (sig.)p < 0.01	↓ (sig.)p < 0.01	↓ (sig.)p < 0.01	↓ (sig.)p < 0.05
		RA patients on anti-TNF-α (n = 10)	BL + after 6 weeks(1 calibrated examiner)	NSp > 0.05	NSp > 0.05	NSp > 0.05	NSp > 0.05	NSp > 0.05
		Anti TNF (n = 10) versus no anti-TNF-α (n = 10)	BL + after 6 weeks(1 calibrated examiner)	NS p = 0.37	↓ (sig.) p < 0.001	↓ (sig.) p < 0.001	NS p = 0.107	↓ (sig.) p < 0.001
Kadkhoda et al. (29)	ETN (n = 36)	RA patients (n = 36)	BL + after 6 weeks	NS *p = 0.860	↓(sig.)p = 0.036	↓(sig.) p = 0.049	NS p = 0.126	–
**3 months follow up**								
Kobayashi et al. (30)	ADA (n = 20)	Patients with RA (n = 20)	BL + after 3 months(2 calibrated examiners)	NS p = 0.12	↓(sig.) p = 0.002	↓(sig.) p = 0.003	↓(sig.) p = 0.002	NSp = 0.42
Kobayashi et al. (31)	IFX (n = 6)ETN (n = 9)ADA(n = 19)Golimumab (n = 6)	RA patients treatment with anti-TNF-α (n = 40)	BL + after 3 months(1 examiner calibrated + masked)	NS	↓(sig.) p < 0.017	↓(sig.) p < 0.017	↓(sig.) p < 0.017	NS
**6 months follow up**								
Kobayashi et al. (31)	IFX (n = 6)ETN (n = 9)ADA(n = 19)Golimumab(n = 6)	RA patients treatment with anti-TNF-α (n = 40)	BL + after 6 months(1 examiner calibrated + blinded)	NS	↓(sig.) p < 0.017	↓(sig.) p < 0.017	↓(sig.) p < 0.017	NS
Savioli et al. (32)	IFX (n = 15)ETN (n = 2)ADA (n = 1)	RA patients with PD (n = 8)	BL + after 6 months(1 blinded examiner)	↓(sig.) p = 0.03	–	NS p = 0.50	NS p = 0.25	NSp = 0.84
		RA patients without PD (n = 10)	BL + after 6 months(1 blinded examiner)	NSp = 0.27	–	NSp = 0.95	NS p = 0.36	NSp = 0.91
Fabri et al. (33)	IFXETNADA	AS patients and PD (n = 7)	BL + after 6 months(1 blinded examiner)	NS p = 0.21	–	NS §p = 0.25	↓(sig.) p = 0.01	↓(sig.) p = 0.04
		Patients with RA and PD (n = 7)	BL and + 6 months(1 blinded examiner)	NS p = 0.076	–	NS §p = 0.118	NS p = 0.381	NSp = 0.36
Ancuta et al. (34)	IFX, ETN and ADA	RA patients (n = 96)	BL and after 6 months	NS p > 0.05	NSp > 0.05	↓(sig.) p < 0.05	↓(sig.) p < 0.05	↓(sig.) p < 0.05
Iordache et al. (35)	IFX, ETN, ADA andGolimumab	AS patients (n = 86)	BL and after 6 months	NS p > 0.05	NSp > 0.05	↓(sig.) p < 0.05	↓(sig.) p < 0.05	↓(sig.) p < 0.05
**9 months follow up**								
Pers et al. (36)	IFX (n = 20)	RA patients with PD (n = 9)	BL + after 9 months(1 blinded examiner)	NS p > 0.05	↑(sig.) † p < 0.05	↑(sig.) ‡p < 0.05	NS p > 0.05	↓(sig.)p < 0.05

RA, rheumatoid arthritis patients; RA+, rheumatoid arthritis with anti-TNF-α therapy; AI, Autoimmune disease; PA, psoriatic arthritis; SSc, systemic sclerosis; AS, ankylosing spondylitis; PD, periodontitis; PDT, periodontal treatment; GI, gingival index; PI, plaque index; BOP, bleeding on probing; PPD, probing pocket depth; CAL, clinical attachment level; IFX, infliximab, ETN, etanercept; ADA, adalimumab; BL, baseline, n, number; sig., statistically significant; NS, not statistically significant, *Oral hygiene index (OHI), ^§^Gingival bleeding index (GBI), ^†^Modified gingival index (MGI), ^‡^Papillary bleeding index (PBI).

Green: significant improvement, Red: significant worsening.

The following results describe studies on TNF inhibitors used for 30 days, 6 weeks, 3 months, 6 months and 9 months. Results are described and compared to baseline measurements and potentially give insight in which parameter improves over time.

#### 30 Days Anti-TNF May Improve PI and GI

Üstün et al. carried out a longitudinal study of 30 days assessing the effect of infliximab and adalimumab in RA patients with periodontitis. 30 Days of anti-TNF-α therapy increased GI significantly while PI, BOP, PPD, and CAL did not change significantly over this time (**Table 2**).

Even in RA patients without periodontitis anti-TNF resulted in an increase of GI (27).

### 6 Weeks of Anti-TNF May Improve PI, GI, and BOP

**Table 2** describes the findings of the study of Ortiz et al. that evaluated the effect of the usage of anti-TNF on periodontal treatment with a follow-up of 6 weeks. Patients on anti-TNF therapy (infliximab, etanercept or adalimumab) were compared to patients not receiving this therapy resulting in improvement of GI, BOP, and CAL when periodontal treatments were conducted. Anti-TNF therapy without periodontal treatment however did not result in improvement of any of the periodontal parameters (28). Another study investigated the effect of anti-TNF medication by administration of etanercept in RA patients for 6 weeks resulted in a significant reduction of GI and BOP while the oral hygiene index (OHI) remained unchanged (29).

#### 3 Months of Anti-TNF Improves GI, BOP, and PPD

Studies with a follow-up period of 3 months are presented in **Table 2**. One of these studies is by Kobayashi et al. (30) and described the effect of adalimumab on periodontal parameters in a group of RA patients (n = 20). Assessments were performed at baseline and after 3 months of administration of adalimumab. Anti-TNF medication decreased GI, BOP and PPD. However no significant changes were observed for CAL. In 2015 Kobayashi et al. (31) carried out another study where the effect of anti-TNF [infliximab (IFX), etenarcept (ETN), adalimumab (ADA), or golimumab] on periodontal parameters was assessed after 3 and 6 months. After 3 months of anti-TNF administration GI, BOP and PPD improved significantly.

#### 6 Months of Anti-TNF Further Improved All Periodontal Parameters Reported 

After 6 months (**Table 2**) the same significant decrease in GI, BOP, and PPD was observed (29).

**Figure 2:**
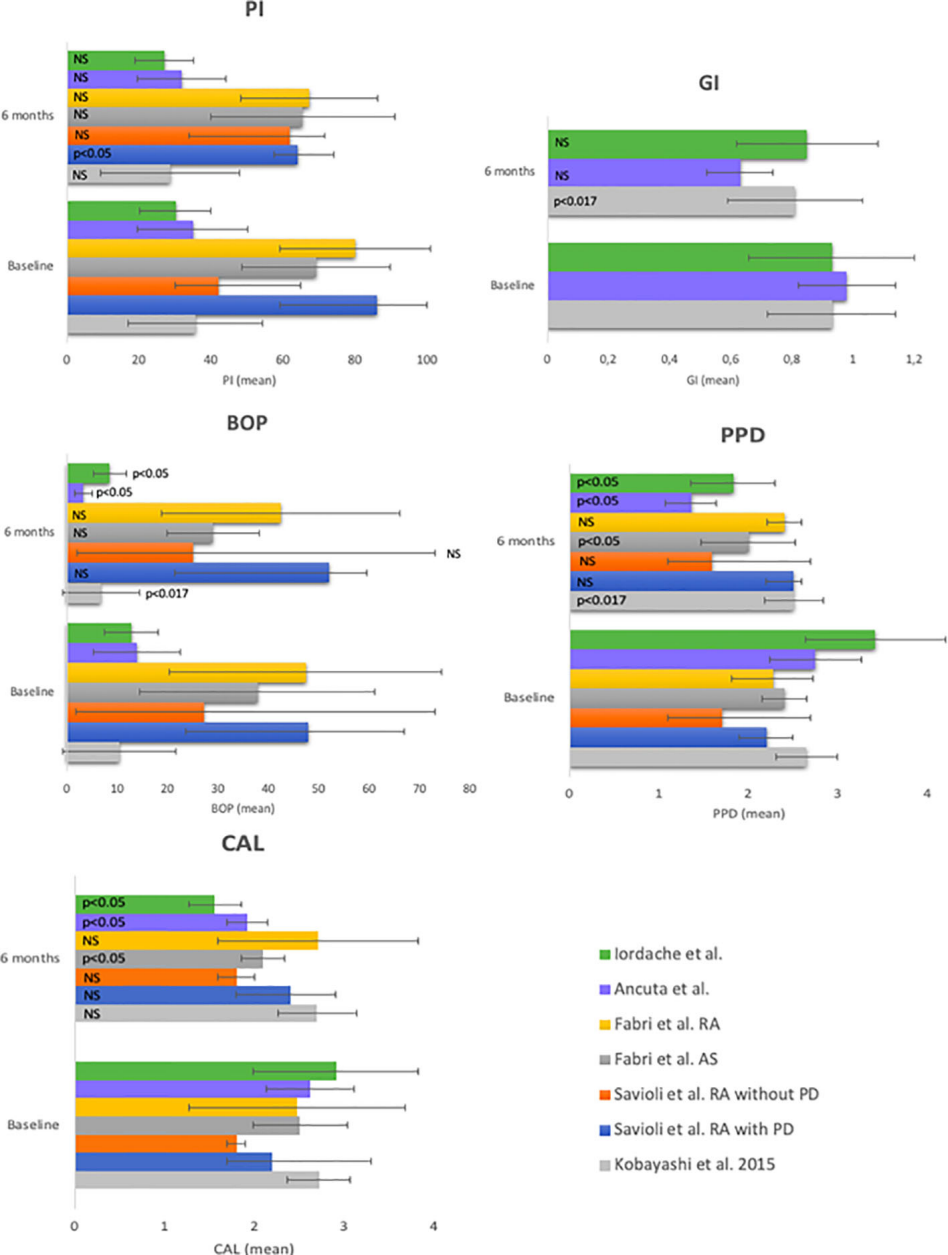
Periodontal parameters at baseline and at 6 months of anti-TNF-α treatment. PI, plaque index; GI, gingival index; BOP, bleeding on probing; PPD, probing pocket depth; CAL, clinical attachment level.

Though not all parameters were analyzed in all studies it is apparent that significant improvements have been reported for all parameters. Savioli et al. tested the effect of the use of TNF inhibitors (IFX, ETN, and ADA) in RA patients with and without periodontitis. In both groups there was no significant improvement in BOP, PPD or CAL. However in the RA group with periodontitis the PI decreased significantly (p < 0.03) (32). Fabri et al. researched the effect of IFX, ETN, and ADA on the periodontal parameters in ankylosing spondylitis (AS) (n = 15) and RA patients (n = 15). AS patients showed an improvement in PPD (p = 0.01) and CAL (p = 0.04) while the other parameters (PI and gingival bleeding index) remained stable (p > 0.05). In the RA group no significant changes were observed (33). Two other studies in this group reached similar results. Ancuta et al. examined RA patients (n = 96) and Iordach et al. examined AS patients (n = 86). Both studies showed a statistical significant decrease after 6 months of anti-TNF therapy in GI, BOP, PPD, and CAL (34, 35). Since the literature has reported most frequently on the 6 months after treatment, the quantitative effects of before and after treatment on the 5 periodontal parameters is summarized in **Figure 2**. Seven studies evaluated the effect on periodontal parameters after administration of anti-TNF treatment for 6 months. The quantitative analysis is shown in **Figure 2**. Three of the seven studies (31, 33, 34) showed a significant decrease in BOP, four studies showed a significant decrease in PPD [(33), in AS patients] and in RA patients (31, 34), and these three studies showed a significant decrease in CAL despite the stability of the PI. This indicates that changes in BOP, PPD, and CAL are not a result of a change in oral hygiene, but rather from systemically tempering TNF.

#### 9 Months of Anti-TNF and Worsening of GI BOP and Improvement of CAL

The final study with a follow up period of 9 months is Pers et al. (**Table 2**). This longitudinal study included 9 patients with RA and periodontal disease. These patients were assessed before anti-TNF therapy with IFX and after 9 months. Results showed that the modified GI (MGI) and papillary bleeding index (PBI) increased, therefore worsened, significantly after 9 months of IFX therapy, while CAL decreased significant. No significant changes were observed for PI and PPD (36).

### Efficacy of TNF Treatment for RA 

In order to control for the efficacy of anti-TNF for the original application, to minimize the rheumatoid arthritis induced inflammation burden, various parameters can be measured. Nearly all studies, except Pers et al. measured the Disease Activity Score 28 (DAS28) (37) and cytokine levels in serum or gingival crevicular fluid (GCF). DAS 28 measures the activity of disease by evaluating the number of swollen and tender joints out of 28. Other parameters were the erythrocyte sedimentation rate (ESR) or C-reactive protein(CRP) and a self-assessment of health using the visual-analog scale (VAS) (37).

By evaluating changes in these parameters, the effectiveness of TNF-inhibitors in reducing inflammation can be evaluated. These findings are shown in **Table 3**.

**Table 3:** Studies assessing the effect of TNF inhibitors on rheumatologic parameters and cytokine levels.

Authors	Subjects	Assesment	DAS28	Serum CRP levels	Serum TNF-α levels	GCF TNF-α levels
Mayer et al. (24)	RA+ (n = 10) compared to RA without anti-TNF-α (n = 10)	1 moment(1 calibrated examiner)	NS p > 0.05	–	–	↓(sig.) p = 0.04
Mayer et al. (25)	RA+ compared to AI (RA, PA and SSc)	1 moment(2 calibrated examiners)	–	–	–	↓(sig.) p = 0.002
Schiefelbein et al. (26)	RA with PD on anti-TNF-α for >12 months (n = 13) versus non RA with PD (n = 13)	1 moment(1 calibrated examiner)	–	NS p = 0.310	–	–
Üstün et al. (27)	RA patients with PD (n = 10)	BL + after 30 days(1 examiner)	↓(sig.) p = 0.001	↓(sig.) p = 0.02	–	–
Ortiz et al. (28)	RA patients on PDT and anti-TNF-α (n = 10)	BL + after 6 weeks(1 calibrated examiner)	↓(sig.) p < 0.005	–	↓(sig.) p < 0.001	–
	RA patients on anti-TNF-α (n = 10)	BL + after 6 weeks(1 calibrated examiner)	NS p > 0.005	–	NS p = 0.2	–
Kadkhoda et al. (29)	RA patients (n = 36)	BL + after 6 weeks	–	–	–	↓(sig.) p = 0.04
Kobayashi et al. (30)	Patients with RA (n = 20)	BL + after 3 months(2 calibrated examiners)	↓(sig.) p < 0.001	↓(sig.) p < 0.001	↓(sig.) p < 0.001	–
Kobayashi et al. (31)	RA patients treatment with anti-TNF-α (n = 40)	BL + after 3 and 6 months(1 examiner calibrated + masked)	↓(sig.) p < 0.017	↓(sig.) p < 0.017	↓(sig.) p < 0.017	–
Savioli et al. (32)	RA patients with PD (n = 8)	BL + after 6 months(1 blinded examiner)	NS p = 0.11	NS p = 0.55	–	–
	RA patients without PD (n = 10)	BL + after 6 months(1 blinded examiner)	↓(sig.) p = 0.04	↓(sig.) p = 0.01	–	–
Fabri et al. (33)	AS patients and PD (n = 7)	BL and + 6 months(1 blinded examiner)	–	↓(sig.) p = 0.03	–	–
	Patients with RA and PD (n = 7)	BL and + 6 months(1 blinded examiner)	↓(sig.) p = 0.01	↓(sig.) p = 0.008	–	–
Ancuta et al. (34)	RA patients (n = 96) RA patients (n = 96)	BL and after 6 months	↓(sig.) p < 0.05	↓(sig.) p < 0.05	↓(sig.) p < 0.05	–
Iordache et al. (35)	AS patients (n = 86)	BL and after 6 months	↓(sig.) *p < 0.05	↓(sig.) p < 0.05	–	–
Pers et al. (36)	RA patients with PD (n = 9)	BL + after 9 months(1 blinded examiner)	–	–	–	–

RA, rheumatoid arthritis patients; RA+, rheumatoid arthritis with anti-TNF-α therapy; AS, ankylosing spondylitis; AI, Autoimmune disease; PA, psoriatic arthritis, SSc, systemic sclerosis; PDT, periodontal treatment; PD, periodontitis; DAS28, disease activity score; CRP, C-creative protein; sig., statistically significant; NS, not statistically significant *ASDAS28 ankylosing spondylitis disease activity score.

Green: significant improvement, Red: significant worsening.

Most of the studies showed a decrease in DAS28, serum CRP levels or TNF levels and thus the anti-inflammatory effect of anti-TNF therapy. A few studies did not show a decrease in these inflammatory parameters. Schiefelbein et al. showed no decrease in serum CRP levels when RA patients on anti-TNF were compared to RA patients without anti-TNF (26). Ortiz et al. also showed no improvement in DAS28 and serum TNF levels in RA patients after 6 weeks on anti-TNFtherapy (28). Furthermore, Savioli et al. showed no significant change in DAS28 and serum CRP levels after 6 months of anti-TNF in RA patients with periodontitis (32).

## Discussion

This literature review presents an overview of the possibly beneficiary effect of TNF inhibitors used in rheumatoid arthritis on periodontal parameters, with special emphasis on which parameters improve over time. The effectiveness of TNF inhibitors in periodontal therapy can be assessed when measuring the anti-inflammatory effects of TNF inhibitors on periodontal parameters of the examined subjects. Measurement of DAS28 is essential in all studies in order to establish effect on joints due to TNF inhibitors in patients. Studies have shown that it takes 2 weeks of anti-TNF (infliximab, etanercept, adalimumab, or golimumab) administration to observe a clinical response in RA and AS patients (38–41). Therefore, a study design with a period shorter than 2 weeks is likely not to show any improvement in periodontal or any inflammatory parameter. In line with this, the study with the shortest standardized period in this review was Üstün et al. with therapy for 30 days which did not show a response of the periodontal parameters (27). This indicates that a possible periodontal response requires more time than an initial dampening of inflammation. Probably, an adequate response for periodontal tissues is shown after 3–4 months of anti-TNF therapy in most patients (42, 43). Ortiz et al. studying the effect of anti-TNF without periodontal treatment for 6 weeks is one of the studies where probably a too short time window was used to observe any adequate response. However, particularly in this study, also DAS28 or serum TNF levels (28) were not altered. This suggests that there is no overall response in these patients to anti-TNF therapy, because either the administration period was too short or there was no response to the medication (primary inefficiency) which is seen in 30–40% of RA patients when using TNF inhibitors (44). On the other hand Ortiz et al. show that patients with RA on anti-TNF therapy demonstrate a significant improvement in GI, BOP, and CAL when anti-TNF therapy was combined with periodontal therapy. Ortiz et al. proved that there is a significant additional value when anti-TNF combined to PDT is used to treat periodontitis compared to patients only receiving periodontal treatment or only using anti-TNF-α. In another study where anti-TNF was administered for 6 weeks, it was found that periodontal parameters (GI and BOP) and cytokine levels (GCF TNF levels) were significantly decreased (29). These changes indicate that anti-TNF leads to less inflammation and some improvement of periodontal parameters, but does not result in a decreased PPD. These observed changes might be a result of anti TNF therapy.

The time point 6 months is the most widely studied, therefore, we summarized only this time point quantitatively (**Figure 2**). Although these 6 studies with 7 different patient groups show significant and beneficiary responses, one should bear in mind that high standard deviations persist which occasionally overlap severely. Besides this, some improvements maybe significant, but many of them are rather moderate.

When summarizing the findings of this study, the periodontal status of rheumatoid arthritis patients receiving anti-TNF administration benefitted from this treatment, especially when analyzed from 6 weeks up to 6 months after start of intervention. However, an administration period of more than 9 months resulted in aggravation of the gingival condition and increase of GI. In rheumatoid arthritis, studies have shown that over time 30–40% of the patients may lose response to anti-TNF treatment (secondary loss of response) (44). Secondary loss of response can be developed by treatment with biological agents. These agents, like anti-TNF therapy can induce an immune response causing formation of ADAs (anti-drug antibodies) resulting in a neutralizing effect of this drug (45). The results by Pers et al., may hint that this also accounts for losing benefit for the periodontal status in these patients. No cytokine levels were measured in this study so nothing can be said about the responsiveness to therapy of the patients in the study group. Scheifelbein et al.^3^ proved that long-term administration (>12 months) still had a positive effect on cytokine levels, and periodontal parameters. Mayer at al. in 2009 (24) and in 2013 (25) also proved that in RA patients with a mean anti-TNF administration of 26 months both GCF cytokine levels and periodontal parameters improved significantly.

As earlier described responsiveness to the anti-TNF therapy has been evaluated per study group in the included studies and not per individual. Lack of responsiveness to anti-TNF therapy might be correlated to ineffectiveness of improving periodontal parameters. Therefore, it is of importance to evaluate if lack of responsiveness in one subject translates in the same subject to a lack of improvement in periodontal parameters. When identifying predictive parameters, anti-TNF therapy could be applied in therapies combating inflammatory diseases. Alternatively, in cases of non-responsiveness, IL-6-R inhibitor treatment could be an option, as described by Kobayashi and coworkers (31). Shortcomings of all studies discussed here, are the relatively small number of included subjects and scarce comparisons between the effects of the different TNF inhibitors. Therefore, the results in this review might show an underestimation or overestimation of some TNF-inhibitors. More research is required to evaluate the effect of each different TNF inhibitor to asses which one is the most effective for the periodontal paramenters. Moreover, no studies have been carried out with healthy individuals with periodontitis on anti-TNF therapy. Such a study could be considered, although one should take into ethical clinical consideration whether one should expose relatively healthy people to a drug that makes the immune system less alert.

Pers et al. (36) showed a significant reduction in CAL but not in PPD. To explain this observation clinical attachment gain, coronal migration of periodontal support, must have occurred and/or the alveolar bone must have increased in height. However this last explanation does not support the findings of Cenk Durmuslar et al. (46), where rats showed no bone regeneration after use of infliximab, which is also in line with Ferreira-Junior et al. (47), where studies in rats proved that infliximab did not affect bone remodeling. Since only one study in this review suggests that there might be a beneficial effect in bone remodeling by the use of anti-TNF there is not enough evidence to support this claim.

**Figure 3:**
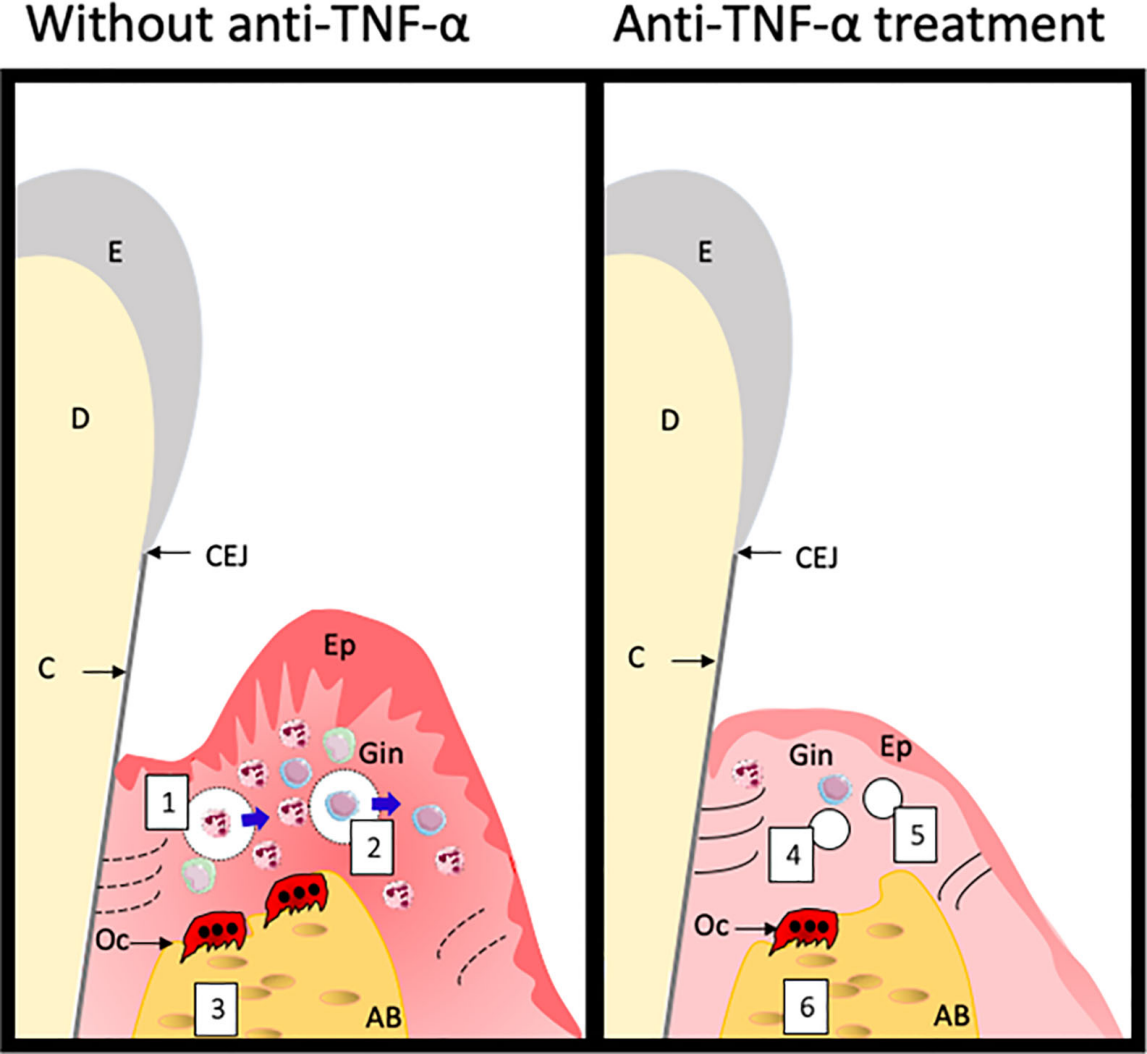
Hypothetical changes of the periodontium following anti-TNF treatment. Treatment with anti-TNF-α leads to overcoming the TNF-α associated cellular processes such as vasodilatation and permeability for leukocytes (1), proteolysis by this excess of leukocytes of extracellular matrix components, depicted as dotted lines (2) and activation of osteoclasts (3). Anti-TNF-α treatment may lead to less dilated and less permeable blood vessels (4), less proteolytic activity, restoring the extracellular matrix production (5) and tempering osteoclast formation and activity. Clinically, this all leads to less bleeding on probing, and an improvement in attachment which is observed by a decreased PPD and CAL. E, Enamel; CEJ, cemento-enamel-junction; C, cementum; D, dentin; Ep, epithelium; Gin, Gingiva; Oc, osteoclast; AB, alveolar bone.

Based on the findings described in this paper and based on general cell biological knowledge of TNF-α (8), we propose the following model, showing the sequence of events that may take place in the periodontium when anti-TNF therapy is effective. TNF has been correlated to processes such as increased PMN rolling on endothelial cells, concomitant with increased classical adhesion molecules for this process such as ICAM-1 and P-selectin, a process that was counterbalanced by anti-TNFtreatment (48). More recently, the effect of TNF on diapedesis showed that especially PMNs migrate into tissues when TNF is high (49). Since an effect on BOP is one of the first aspects that improves, we would like to suggest that vessel permeability is first of all restored, together with a downregulation of endothelial adhesion molecules for diapedesis, such a ICAM-1 (**Figure 3**, numbers 1 and 4). This then results in a decrease in leukocytes that migrate into the tissue (**Figure 3**, numbers 2 and 5). Since these leukocytes are potential producers of TNF-α, that may induce proteolytic activity (50) and signaling towards osteoclasts (9, 10, 12, 18), the overall switch is towards anabolic activities (**Figure 3**, numbers 3 and 6). Clinically, this leads to improved PPD and CAL (**Figure 3**). Proof of these hypothetical changes should come from histological assessments, but these are scarce in the field of periodontitis.

This overview presented promising results that anti-TNF therapy is beneficial in the treatment of both RA and periodontitis by improving periodontal parameters of RA patients. One would like to be able to predict which patients with both RA and periodontitis would benefit from which TNF inhibitor. Future research is needed to elucidate this further.

## Author Contributions

Idea for the systematic review: TV. Literature search and first draft: FZ. Writing: FZ and TV. Completion: FZ and TV. All authors contributed to the article and approved the submitted version.

## Conflict of Interest

The authors declare that the research was conducted in the absence of any commercial or financial relationships that could be construed as a potential conflict of interest.
